# Periphery-Brain Interactions and Leptin in the Regulation of Whole-Body Energy Metabolism

**DOI:** 10.3390/nu14081594

**Published:** 2022-04-12

**Authors:** Mohammed Khair Hankir, Michael Bruneau

**Affiliations:** 1 Department of Experimental Surgery, University Hospital Würzburg, 97080 Würzburg, Germany; 2 Department of Health Sciences, Drexel University, Philadelphia, PA 19102, USA

In order to combat overweight and obesity as a global public health issue and prevent its impact on other debilitating cardiovascular, metabolic and renal diseases, a better understanding of the processes regulating energy metabolism are essential. Our aim in curating the current Special Issue on “Periphery-Brain Interactions and Leptin in the Regulation of Whole-Body Energy Metabolism” was to provide readers of Nutrients and scientific researchers within the nutrition, energy metabolism and bariatric communities with a richer resource on how periphery-brain interactions and leptin signaling can be targeted to develop innovative new pharmacologic and non-pharmacologic treatments for various metabolic diseases. To successfully achieve this aim, we attracted researchers to contribute scientific publications across the extremes of the adiposity spectrum, from anorexia nervosa and lipodystrophy to morbid and severe overweight and obesity.

Hankir et al. [1] revisited the premise that enhanced central leptin sensitivity contributes to the beneficial effects of RYGB on body weight status, glycemic control, and liver health. The researchers performed RYGB on Zucker fatty rats that developed overweight and obesity, glucose intolerance and fatty liver disease from functional mutation loss in the leptin receptor gene and diet-induced obese rats with intact leptin receptors. It was found that weight loss after RYGB in both strains of rats were largely similar, further supporting the premise that leptin receptors are dispensable for the effects of surgery on energy metabolism. Moreover, food intake in otherwise hyperphagic Zucker fatty rats was completely normalized by RYGB to the status of lean controls, suggesting that surgery modifies brain feeding circuits in a leptin receptor-independent manner. Oral glucose tolerance was also improved in Zucker fatty rats after RYGB, further supporting that leptin receptors are dispensable for the effects of surgery on glucose metabolism. Interestingly, in a preliminary analysis of liver function, the liver enzyme alanine aminotransferase remained elevated in Zucker fatty rats after RYGB, suggesting that leptin receptors are required for the improvement of liver health after surgery. These findings suggest that when developing drugs mimicking the effects of RYGB on energy and glucose metabolism, leptin sensitization may not be necessary. However, when developing drugs that mimic the effects of RYGB on fatty liver disease, leptin sensitization may be necessary.

Stable analogues of the gut hormone glucagon-like peptide 1 (GLP-1) such as liraglutide and semaglutide are currently used for the treatment of overweight and obesity and cause significant weight loss in obese patients largely via the reduction of food intake. However, it remains incompletely understood how liraglutide affects the central nervous system. To address this question, Dischinger et al. [2] performed RNA sequencing on hypothalamic samples taken from diet-induced obese rats receiving liraglutide alone, liraglutide in combination with the gut hormone peptide tyrosine tyrosine (PYY), RYGB or chronic caloric restriction for four weeks. Strikingly, while RYGB and chronic caloric restriction had a profound impact on the hypothalamic transcriptome and upregulated overlapping signaling pathways [2,3], gut hormone treatments had virtually no effects. Despite their preclinical nature, these findings have major implications on how long-term treatment with gut hormones affects brain health.

Patients with lipodystrophy, a congenital or acquired condition characterized by a lack of adipose tissue, suffer from many of the ailments associated with obesity such as fatty liver disease and insulin resistance. Leptin supplementation is used to treat lipodystrophy and has wide-ranging metabolic benefits, but the mechanisms involved remain unclear. Hoffmann et al. [4] asked whether brown adipose tissue thermogenesis is enhanced in response to chronic leptin treatment in a mouse model of congenital lipodystrophy and found that leptin-treated lipodystrophic mice had increased core-body temperature and molecular markers of thermogenesis in brown adipose tissue, such as the mitochondrial protein uncoupling protein 1. Mechanistically, evidence was obtained of increased sympathetic nerve innervation of brown adipose tissue by leptin treatment suggesting that the adipokine uses a similar mechanism as cold exposure to increase thermogenesis. These findings provide a potentially new explanation for how leptin improves glucose and lipid metabolism in otherwise hyperglycemic and hyperlipidemic lipodystrophic patients, since brown adipose tissue consumes large amounts of these nutrients during thermogenesis. These findings also raise the intriguing possibility that lipodystrophic patients might benefit from drugs that more potently stimulate brown adipose tissue thermogenesis than leptin, such as the selective beta 3 adrenergic receptor agonist mirabegron.

In accordance with the premise of targeting pharmacologic and non-pharmacologic strategies for the prevention, treatment and management of “Periphery-Brain Interactions and Leptin in the Regulation of Whole-Body Energy Metabolism”, Nasser et al. [5] investigated the effects of a low (1.2 g) and high (8.0 g) dietary protein-preload and habitual physical activity on dietary intake of ultra-processed high sugar/high fat/low protein food in 50 healthy participants. The premise of the intervention is grounded in the phenomenon of “loss of control”, which Dr. Nasser notes is a major contributor of overweight and obesity. Previous clinical trials have attempted to explore the effects of diet and exercise as non-pharmacologic lifestyle therapies for the prevention, treatment and management of overweight and obesity, and few have explored their effects in an interdependent manner as Altayyar et al. [6] reported in the current investigation. Dietary protein has long been established in the scientific literature as a macronutrient promoting satiety; however, little attention has been given to its effect on reducing subsequent food intake of ultra-processed low-protein snack foods, and none have considered whether habitual physical activity moderates these effects in a dose-dependent manner. The authors found that 30 of the 50 (60%) participants in the study sample were responders to the protein pre-load, operationalized as those who reduced their intake of ice cream, an ultra-processed high sugar/high fat/low protein food, after consuming an 8 g protein preload. This finding supports the authors’ central hypothesis that a high protein preload consumed near an ultra-processed high sugar/high fat/low protein food can be reduced as compared to a low protein preload. Interestingly, the authors found that responders and non-responders’ physiological response to each protein preload was not dependent upon self-reported habitual physical activity; however, among non-responders, those who engaged in more habitual physical activity consumed less ice cream ad libitum, consistent with previous physical activity and exercise studies exploring the interdependency between energy intake and energy expenditure. Furthermore, the authors concluded that protein consumed as a preload to ultra-processed food intake can produce clinically meaningful reductions in caloric intake at the magnitude of ~60 kcals per episode, which could potentially prevent the likelihood of body weight gain of at least 5 lbs. per year.

In recognition of novel dietary approaches for the treatment and management of overweight and obesity, Altayyar et al. [6] published a narrative review on the implications of physiological ketosis on the cognitive brain. The authors narrated that regressive changes in cognitive function, perhaps as a proxy of overweight and obesity, advanced physiological age, or their intersection, can be mitigated with various dietary and nutritional approaches. The authors highlight the importance of glucose as a core macronutrient and biosource of energy that may be affected from aging neurons or from other developments from chronic disease. The authors thereafter highlight ketone bodies as an efficient fuel source that can feasibly compensate for deficient glycolytic metabolism. Encompassed within their review, the authors describe the premise and potential for ketogenic diets and their purported benefits on cognitive function in adults free from cognitive deficits and disease, those living with mild cognitive impairment (MCI), and those living with Alzheimer’s disease and related dementias (ADRDs). The authors further review current neurophysiological changes reported from the literature on the cognitive brain’s response to physiological ketosis in neuroimaging studies and provide additional insight into the ketogenic effects of the diet on brain function at large. Of clinical importance, Altayyar et al. narrate that ketogenic diets have potential for releasing several anti-inflammatory cytokines, which are paramount for reducing one’s risk for cognitive health issues such as MCI and ADRDs in people across their lifespan; however, recognition of the importance for weight loss, independent of a ketogenic diet, is also anti-inflammatory and should be considered. The authors also review important preliminary evidence regarding the use of medium-chain triglycerides and intermittent fasting as strategies to induce nutritional ketosis, but recognize these studies as being in their infancy with a need for additional trials to substantiate their long-term safety, efficacy and relationship with other risk factors for cardiovascular, metabolic and renal diseases as important areas of investigation for the future.

The articles in this Special Issue shed light on the multifaceted effects of leptin and other interventions on energy metabolism and how neuroactive molecules like ketone bodies, typtophan metabolites, and chemokines impact cognition and metabolic health. Going forward, we hope that this Special Issue provides a useful reference for future studies in the field of periphery-brain interactions and leptin in the regulation of whole-body energy metabolism so that innovative new pharmacologic and non-pharmacologic treatments for various metabolic diseases can be developed.
